# Effect of Embryonic Cerebrospinal Fluid on Proliferation and Differentiation of Neuroprogenitor Cells

**Published:** 2013-05-05

**Authors:** Siamak Yari, Kazem Parivar, Mohammad Nabiuni, Mohammad Keramatipour

**Affiliations:** 1 Department of Developmental Biology, Faculty of Biological Sciences, Kharazmi University, Tehran, Iran; 2 Department of Medical Genetics, Faculty of Medicine, Tehran University of Medical Sciences, Tehran, Iran

**Keywords:** Embryonic Cerebrospinal Fluid, Neuroprogenitor Cells, Proliferation, Differentiation

## Abstract

**Objective:**

Embryonic cerebrospinal fluid (e-CSF) has an important role in development of embryonic and adult brain. Proteomic analysis suggests that this fluid has many morphogenes and cytokines that alter in time and space throughout embryonic life. The aim of this study was to evaluate the developmental effect of embryonic CSF on proliferation and differentiation of neuroprogenitor cells in different gestational age.

**Materials and Methods:**

In this In this experimental study, we examined the role of e- CSF on proliferation and differentiation of neuroprogenitor cells using neurosphere culture method. Neurospheres size analysis and MTT assay were used to assess cell proliferation after four days *in vitro*. Glial differentiation study was carried out by immunocytochemistry. Neurospheres size and percentage of glial fibrialy acidic protein (GFAP) positive cells were measured by image analyzer (image J). The data were analyzed by one-way ANOVA, followed by the Tukey’s post hoc test. Data were expressed as mean ± SEM, and differences were considered significant when p<0.05, 0.01 and 0.001.

**Results:**

Viability and proliferation of neuro progenitor cells in cultures conditioned with E16 CSF and E18 CSF were significantly increased compare to control group. A dramatic decrease in percentage of GFAP-positive cells was found following the application of CSF from E16 and E18 embryos, but not E20 CSF.

**Conclusion:**

Our data suggest that, e-CSF altered proliferation and differentiation of neuro progenitor cells in age dependent manner. E16 and E18 CSF enhanced proliferation and viability of neuro progenitor cells, and inhibited differentiation to glial fate in comparison with control group.

## Introduction

Neural stem cell expansion and cell fate determination are regulated by two intrinsic and extrinsic factors. Intrinsic factors include transcription factors, chromatin remodeling and micro RNAs. The extrinsic factors include extracellular signaling molecules, such as FGFs, WNTs, SHH and BMPs (1). The proteomic analysis of embryonic cerebrospinal fluid (e-CSF) shows that it contains lots of proteins and trophic factors, such as growth factor, extracellular matrix, lipoproteins, and glucose aminoglycans (2). The evidences have showed that e-CSF has membranous particles carrying the stem cell markers and may have a role in self renewal capacity of neuro epithelial cells (3). The neuroepithelial cells, as a batch in CSF, have self renewal capacity and are in close contact with signaling substance, which are present in CSF, thus CSF is considered as an extrinsic signaling source which affects neuro progenitor cells behavior (4). The CSF production after neural tube closure depends on two groups of cells. The first group is neuro epithelial cells lining the wall of neural tube, while the second one is special mesenchymal derived from epithelial cells that invaginate inside the ventricle, named as choroid plexus. During late embryonic developmental period, CSF is largely produced by the choroid plexus, a highly vascularised epithelium found in the lateral, third and fourth ventricles of the brain (5). The recent findings have demonstrated that CSF is involved in early brain development by two mechanisms, hydrostatic pressure and neurotrophic effect (6). In early stages of embryonic brain development, the expansion occurs via CFS accumulation within ventricles. The hydrostatic pressure is produced by this accumulation, which creates tension in neuro epithelium and may enhance proliferation via stimulating the tension receptors (7). Another suggested mechanism is its trophic effect on neuro epithelium. Miyan et al. (8) have hypothesized that neurotrophic factor of CSF has a vital role in brain development. In some neurodegenerative diseases, such as hydrocephalus and multiple sclerosis, disruption of normal CSF flows, so the up-regulation of some morphogenes cause severe abnormalities (9). The cerebrospinal fluid, containing chemo repulsive Slit proteins, has regulatory effect on migration of neuroblast chain from SVZ to olfactory bulb (10). The recent investigations have showed that chicken embryonic CSF enhances survival, proliferation, and differentiation of neuroepithelial cells (11, 12). Embryonic CSF during critical stage of brain development contains membrane particle that carries stem cell marker prominin-1. The presence of this marker in e-CSF is sign of high proliferative behavior of brain neuro progenitors (3). Also, recent findings have revealed that the raised level of this marker in CSF of glioblastoma patient can be used as a diagnostic factor for brain tumors detection (13). The aim of this study was to evaluate the developmental effect of embryonic CSF on proliferation and differentiation of neuro progenitor cells in different gestational age.

## Materials and Methods

### Animals

In this experimental study,Wistar rats, bred in house, were received from the Research Facility of the Faculty of Biological Sciences, Kharazmi University, followed by obtaining the ethical approval from the Animal Uses Committees of Kharazmi University. They were kept in large rat boxes at constant temperature and a 12/12-hour light/dark cycle with free access to food and water. Individual male and female rats were paired in mating cages and checked regularly for the presence of a vaginal plug, which was taken as an indication of successful mating, and the day was noted as embryonic day 0 (E0). Embryonic age was calculated from E0. At a particular time point, pregnant dams were euthanized by intra peritoneal injection of an overdose of sodium pentobarbitone. After rapid removal of the uterine, they were placed on ice, while the fetuses were dissected out. Each pregnant dam usually produced between 10-15 fetuses

### CSF collection

CSF was collected from the cisterna magna of rat fetuses at E16, E18, and E20 using glass micropipettes and capillary action without aspiration. Aspiration invariably resulted in bleeding and contamination of the samples. Fetuses were positioned with heads flexed down onto the chest to allow penetration into the cisternal cavity through the skin and underlying muscle. Samples containing undesirable blood contamination, visualized as a pink color in the fluid, caused by damaging a blood vessel within the cisternal cavity, were discarded. All samples were collected into sterile microtubes and centrifuged at 14,000 rpm to remove cells or debris from the fluid, and the supernatant was transferred into another sterile tube. These samples were stored at -80˚C until use. The volume of CSF collected from each fetus by this method was between 5 and 50 µl, and samples were then pooled for each experiment.

### Neurosphere culture

Neurosphere were prepared as described previously (14). Briefly, pregnant Wistar rat with gestational age of 15.5 days (E15.5) were killed via intra peritoneal injection of an overdose of sodium pentobarbitone. Embryos were removed from the amnion, and the heads were dissected using tweezers. After removal of the overlying meninges and blood vessels, sub ventricular zone (SVZ) was dissected out and transferred to serum-free media. Tissue samples were dissociated using three ml of 0.25% trypsin-ethylene diamine tetraacetic acid (EDTA) solution (Gibco-Invitrogen, CA, USA) at 37˚C, followed by five ml of trypsin inhibitor solution. Cells were centrifuged at 1000 rpm for five minutes, re suspended in five ml of trypsin inhibitor solution, and mechanically dissociated with a fire-polished pipette. Cells were then suspended in five ml of basal media, centrifuged, re suspended in two ml of basal media, and used for experiments. The cells were added to 25-cm2 flasks and maintained in serum-free media comprising Dublecco’s Modified Media (DMEM)/F-12 medium (Gibco-Invitrogen, CA, USA) supplemented with 2% N_2_ (Gibco-Invitrogen, CA, USA) supplement, 1% penicillin/streptomycin (Gibco-Invitrogen, CA, USA), and 20 ng/ml epidermal growth factor (EGF) (Gibco-Invitrogen, CA, USA), 20 ng/ml basic fibroblast growth factor (b- FGF) (Gibco-Invitrogen, CA, USA). Cultures were incubated at 37˚C in a humidified atmosphere and 5% CO_2_, 92% N_2_, and 3% 6 2% O_2_. Fresh medium and growth factors were supplemented every two days

The cultures were divided into following four study groups: i.control: No CSF exposure, ii. E16: exposure to CSF of E16, iii. E18: exposure to CSF of E18 and iv. E20: exposure to CSF of E20. For differentiation studies, after four days *in vitro*, cells obtained by neurosphere splitting were counted, seeded into poly-L-lysine coated-surfaces dish (Sigma-Aldrich, Switzerland), and grown as a monolayer in the same medium without mitogens in order to allow them to differentiate. After four days *in vitro* (DIV) cells were processed by immunocytochemistry in order to perform antigen expression and morphological analysis.

### MTT assay

Cell growth and viability were also studied by the MTT assay (Merck, Germany), a biochemical approach, which was based on the reduction of MTT (3-[4,5-dimethylthiazol-2-yl]-2,5-diphenyl tetrazolium bromide) into formazan crystals by the action of the mitochondrial de hydrogenase enzymes, were present in viable cells. The crystals formed were then dissolved in acidified isopropanol, giving a spectrophotometrically measurable purple solution. Neurospheres were treated with a solution of five mg/ml MTT. After two hours at 37˚C , the formed formazan crystals were dissolved in a solution consisting of 10% Triton X-100/0.1N HCl/ isopropanol, then incubated for one hour at RT in the dark. Absorbance was read at a wavelength of 570 nm. All experiments were carried out in duplicates.

### Immuno cytochemistry

Cells were fixed for 20 minutes in 4% paraformaldehyde containing phosphate-buffered saline (PBS) (pH=7.4), washed in PBS and permeabilized for five minutes with PBS/0.5% Triton X-100 (Merck, Germany). Adherent single cells were incubated overnight at 4˚C in PBS containing 5% bovine serum albumin (BSA) and the appropriate mixture of antibodies. Primary antibody used was rabbit polyclonal anti-GFAP (1/600, Abcam, England) for astrocytes. After washing in PBS, differentiating cells obtained from spillited neurosphere were incubated for one hour with Cy3- conjugated secondary antibodies (1/300, Abcam, England). Nuclei were counterstained with propidiom iodate (1/15000, Sigma-Aldrich, USA).

### Morphometric analaysis

After four DIV in proliferation condition, digital images of the neurospheres cultures were taken using an inverted microscope (Biomedica, China). The magnification of the image (×10) covered a significant area of each well from 24 well plates. An image analysis program (image J) was used to analyze the size of neurospheres. After four DIV in differentiation condition, ten non-overlaping fields were randomly selected from each well, and images were captured using a fluorescence microscopy (Olympus, Tokyo, Japan). Randomly chosen field were counted, and percentage of GFAP-positive cells was determined. All experiments were carried out in duplicates.

### Statistical analysis

Data are presented as the mean ± standard error of the mean (SEM). Statistical analysis was performed using the one-way ANOVA and Tukey’s post hoc test, and significance was accepted for p values of <0.05.

## Results

**Fig 1:**
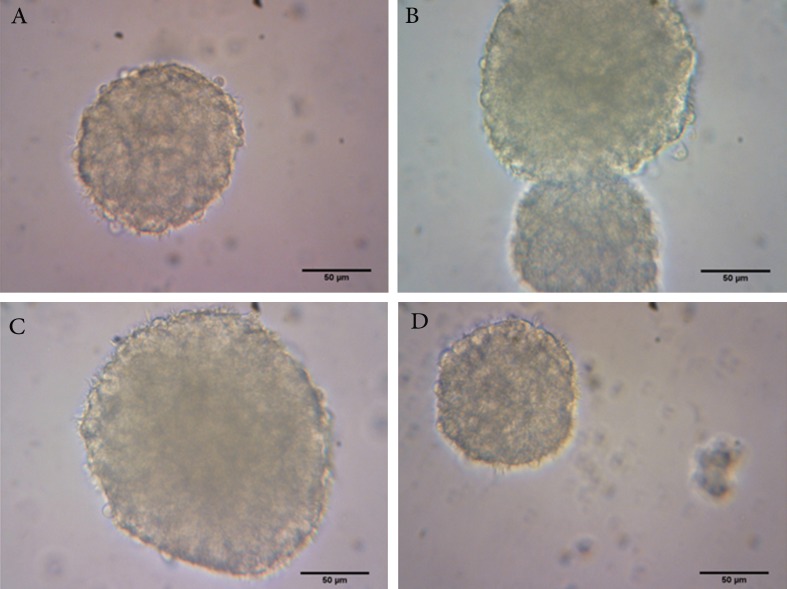
Photomicrographs of neurospheres were cultured in presence of CSF from E16 (B), E18 (C), E20 (D) and control (A). Photomicrographs were taken at magnification ×400.

**Fig 2:**
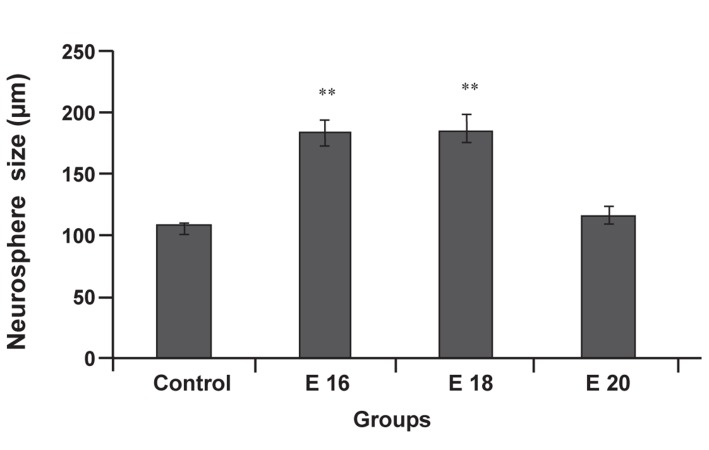
Effect of e-CSF on neurosphere size. Statistically significant differences between treated groups (E16 and E18) were detected compared with control group. Data are presented a means ± SEM (**p<0.01).

**Fig 3:**
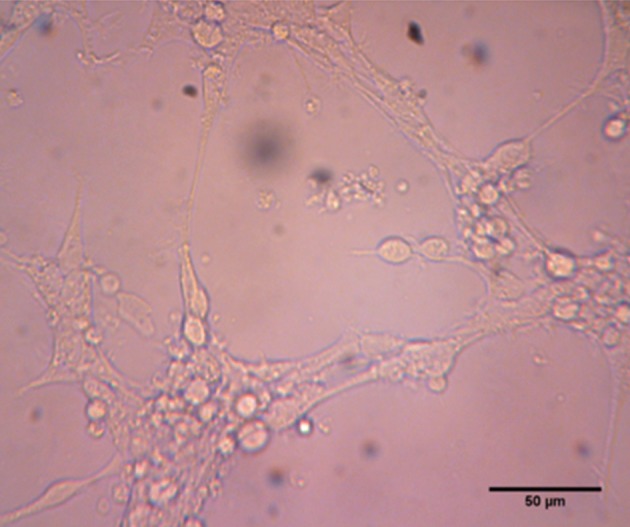
Effect of high ratio e-CSF on adherence. Higher concentration (>10 V/V) of CSF from E16 and E18 are due to enhance the adherence of neurosphere to non-coated culture dish.

In this study, we examined the effect of e-CSF on proliferation and differentiation of neuroprogenitor cells. Increasing in the size of neurosphere was considered as a sign of increasing in proliferation rate of neuroprogenitor cells. As shown in figures 1 and 2, significant increase in the size of neurosphere were detected in groups treated with CSF from E16 (186.35 ± 11.37, p<0.01) and E18 (190.7 ± 11.65, p<0.01) in comparison with control (109.26 ± 4.26). No obvious differences between culture treated with CSF from E20 and control were observed. Our results showed adding CSF to culture medium caused differential effects on growth characteristics and morphology of neuroprogenitor cells. The media was immediately treated with 10% of CSF after cell seeding established sphere forming characteristic. When CSF was added to medium in high ratio, the neuroprogenitor cells showed adherent characteristic and began to differentiate (Fig 3).

Measurement of neurosphere size in different cultures condition (in presence and absence of e- CSF) provided interesting results. E16 (Figs 1B, 2) and E18 (Figs 1C, 2) CSF-treated-neurospheres were significantly greater than neurosphere of control group (Fig 1A, 2). But, E 20 CSF-treaded neurospheres (Fig 1D, 2) did not show any significant difference from neurospheres of control group.

**Fig 4:**
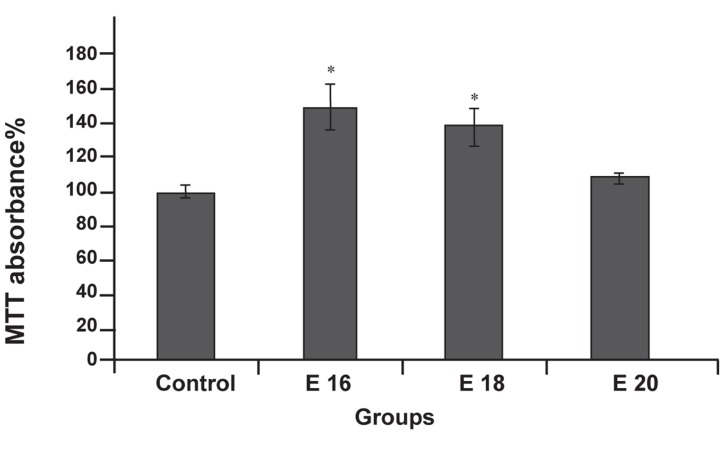
MTT reduction activity of neurosphere culture conditioned with e-CSF and control. Data are expressed as a percentage of control levels (cultures without added CSF). Culture conditioned with CSF from E16 and E18 had significant viability and proliferation compared to control. Data are presented as mean ± SEM (*p<0.05).

To examine the effects of e-CSF on the survival and proliferation of neurospheres, MTT assay was used to quantify cell proliferation and viability. It is noted that in this technique, the MTT conversion relies on the ability of the viable cells to reduce a water soluble yellow dye to a water-insoluble purple formazan product. Each absorbance value of MTT reduction activity was normalized by the results of control group. The MTT conversion of neurospheres in the presence of e-CSF from E16 and E18 was significantly higher than control group (p<0.05, Fig 4)

**Fig 5:**
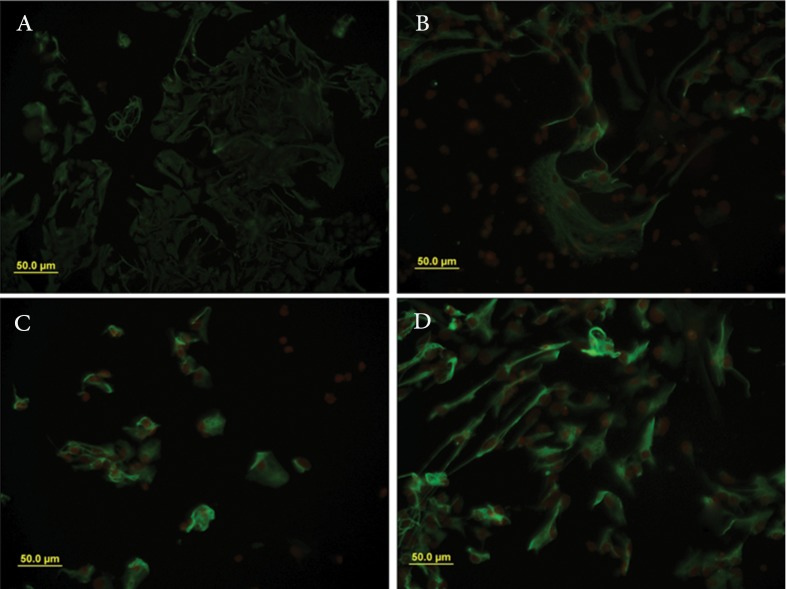
Immunocytochemical analysis of GFAP. Photo-micrographs of immuonopositive cells in groups treated with CSF from E16 (B), E18 (C) and E20 (D) and control.

**Fig 6:**
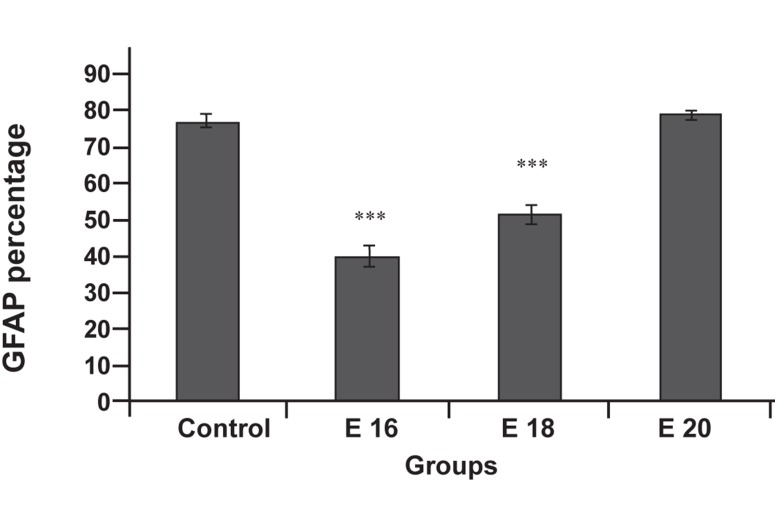
Effect of e-CSF on neuroprogenitors cells differentiation. GFAP positive cells in culture conditioned with e- CSF, while in control were evaluated by an image analysis. Statistically significant differences between treated groups (E16 and E18) were detected compared with control group (***p<0.001).

These results suggest that e-CSF may play an important role in regulating the differentiation of neural progenitor cells. We examined whether the addition of e-CSF, isolated from different embryonic age, could modulate the number of differentiated neuroprogenitors. Upon differentiation of neuroprogenitor cells through exposure to serum on a poly-L-lysine coated dish, we found out that addition of E16 and E18 CSF strongly decreased differentiation of adherent neuroprogenitor cells to glial fate. E16 (39.93 ± 2.83, p<0.001) and E18 (51.67 ± 2.84, p<0.001) CSF-treated media were shown to cause significant decrease in percentage of GFAP-positive cells in comparison with control group (77.16 ± 2.10). It was not increased over controls in CSF from E20 (79.01 ± 1.11, Fig 5, 6).

## Discussion

We have investigated the regulatory effect of CSF *in vitro* on neuroprogenitor cells behavior using rat embryos of different gestational ages. It seems that e-CSF neurotrophic component modulates proliferation and differentiation of neuroprogenitor cells in age dependent manner.

This study demonstrated e-CSF involvement in the proliferation of neural progenitor cells. e-CSF enhances proliferation of neuroprogenitors after placing them in non-adhesive culture dish, also it significantly increases neurosphere size compared to control group. It has been reported previously that e-CSF has mitogenic effect on neural progenitor cells isolated from the embryonic brain of chicken and rat with different culture method (12-15). In this study, we demonstrated that e-CSF enhances neural progenitor cells proliferation in neurosphere culture method. Previous study has showed that neurospheres have heterogeneous populations of cells in different committed state along with neural lineages (14). Also, analysis of e- CSF shows that different mitogenic factors with various concentrations are present in this fluid (16). So, we conclude that these distinct populations of neuroprogenitors have potential to differentiation in response to e-CSF. Findings suggested that soluble form of amyloid precursor protein (sAPP) is present in CSF (17). In addition, *in vitro* studies have showed that this component enhances proliferation of embryonic neuroprogenitor cells (18, 19). Also, sAPP regulates proliferation of EGF-responsive cells in adult neurogenesis regions such as, SVZ and neuroprogenitors cells, located in binding site of sAPP (20). Since sAPP is present in CSF, further studies are necessary to understand the involved mechanisms in modulation and interaction of sAPP with neuroprogenitors in time and space. Moreover, recent findings have suggested that CSF from chicken embryonic contains lipoproteins and membranous particles that may regulate signaling processes involved in development of embryonic brain (16).

The data suggested that e-CSF modulates differentiation of neuroprogenitor cells to GFAP positive cells in age dependent manner, while it decreases during early rat embryonic life at E16 to E20. It has been supported by *in vivo* studies that astrocytes are immuno-reactive to GFAP, first detected at E16 (21). Recent studies have demonstrated that adult CSF from human and rat has stimulating role in proliferation and viability of neural stem cells. Also, adult CSF promotes differentiation of neural stem cells to glial fate (22). The evidences have showed that central nervous system development is temporarily regulated during early embryonic period by extrinsic factors, such as cell to cell intraction, paracrine factor within brain tissue and factors that originate from outside sources (meninges and CSF) (4). Our results demonstrated that a primary neurosphere conditioned with CSF from rat embryos of different ages has temporal pattern in number of GFAP positive cells. These results are supported by the *in vivo* findings of Liu et al. (21) about GFAP immunostaining cells, during development of embryonic rat brain, which rose in late gestational days. Additionally, Sancho-Tello et al. (23) have demonstrated that radial glial cells cultured in presence of horse serum show increasing level of GFAP in time dependent manner. Furthermore, proteomic analyses show differences between rat e-CSF samples collected from various embryonic ages (2). Growing evidences suggest that CSF plays important role as a stem cell niche and provides a microenvironment with specific features, like containing diffusible signals, acting in gradient manner and regulating neuroepithelial cell proliferation and differentiation (4). The abnormal CSF flow maintains in some disease, such as hydrocephalus and spinabifida, which results in disruption of CSF-borne diffusible signals and affects on normal neurogenesis pathways in this pathologic states (24). Also, regulation of CSF-born growth factor maybe involve in some neurodegenerative disease, such as MS and Alzheimer’s disease. The elevated levels of Ab1-42 in CSF were used as a diagnostic marker in Alzheimer’s disease (25).

## Conclusion

Our result demonstrated that e-CSF altered proliferation and differentiation of neuroprogenitor cells during embryonic period. E16 and E18 CSF enhanced neurospheres sizes as sign of proliferation, and inhibited differentiation to glial fate in comparison with control groups. Since CSF proteomic composition was altered throughout brain developmental processes and in neurodegenerative state, modulation of CSF components could provide a complementary therapeutic method to regulate the neuroprogenitor cells behavior *in vivo* and *in vitro*. Thus, further studies seem necessary to investigate CSF components and synergistic effects of them.
